# Commercial Goat Cheeses Show No Differences in Postprandial Satiety During Caloric Restriction: A Randomized Controlled Pilot Trial

**DOI:** 10.3390/nu18193215

**Published:** 2026-09-29

**Authors:** Diana A. Vela-Vásquez, Ivan Delgado-Enciso, Aram Y. Rubio-Reyes, Estela Garza-Brenner, Claudia P. Beltrán-Altamirano, Ana M. Sifuentes-Rincón

**Affiliations:** 1 Laboratorio de Biotecnología Animal, Centro de Biotecnología Genómica, Instituto Politécnico Nacional, Reynosa 88710, Mexico; dvelav1600@egresado.ipn.mx (D.A.V.-V.); arubior2300@alumno.ipn.mx (A.Y.R.-R.); cbeltrana2400@alumno.ipn.mx (C.P.B.-A.); 2 Facultad de Medicina, Universidad de Colima, Colima 28040, Mexico; ivan_delgado_enciso@ucol.mx; 3 Instituto Estatal de Cancerología de los Servicios de Salud del Instituto Mexicano del Seguro Social para el Bienestar, Colima 28090, Mexico; 4 Laboratorio de Biotecnología y Biología Molecular, Departamento de Ciencias Básicas, Universidad Autónoma Agraria Antonio Narro, Saltillo 25315, Mexico; egbrenner@hotmail.com

**Keywords:** goat cheese, post-meal satiety, weight loss, satiating foods, VAS, acylated ghrelin

## Abstract

**Background/Objectives**: Satiety and appetite control are key strategies for successful weight loss. Goat dairy may increase satiety, highlighting its potential role in weight control and metabolic health. This study evaluated satiety, changes in acylated ghrelin concentrations, and anthropometric outcomes in volunteers following a calorie-deficit diet that included goat cheese with different fat contents. **Methods**: In this randomized controlled clinical trial, volunteers consumed a breakfast containing 80 g of two commercial cheeses differing mainly in their fat content. Satiety perception was assessed using a Visual Analogue Scale at baseline (T0), 30 (T30), and 120 (T120) minutes after breakfast. Acylated ghrelin levels were measured by ELISA at baseline and at the end of the intervention at T0 and T30. **Results**: A reduction in goat cheese fat content (25.9%) did not modify the induction or maintenance of post-meal satiety during energy restriction. Both groups experienced reductions in body weight and anthropometric measurements, with no significant differences between groups. The anthropometric response was predominantly attributable to the energy-restricted intervention and was not significantly modified by the fat content of the assigned goat cheese. Across the intervention, sustained satiety increased modestly over time. Each additional week was associated with an estimated 0.164-point increase in ΔCSI_0–120_ (β = 0.164; 95% CI: 0.058–0.270; *p* = 0.003), similarly in both groups. Complementary analyses confirmed comparable cumulative post-meal satiety responses. **Conclusions**: Sustained satiety improved progressively regardless of goat cheese fat content. However, low-fat cheese may offer a practical benefit for meal planning, providing fewer calories while offering the same filling effect as high-fat cheese.

## 1. Introduction

Satiety is defined as a physiological response that inhibits further eating, reduces hunger, and delays the next meal [1,2]. It plays an important role in energy consumption; therefore, satiety can be useful for interventional purposes since it can act directly by decreasing the frequency of consumption or indirectly by decreasing the size of the next meal [3]. Hansen et al. [4] reported in a meta-analysis that when volunteers were exposed to satiety-enhancing and/or hunger-reducing foods, they showed a decrease in body weight of 3.60 kg compared with controls.

Different studies have shown how dietary components such as high-quality protein, dietary fiber, whole grains, legumes, nuts, and minimally processed foods have the capacity to enhance postprandial satiety [5,6,7] through different physiological pathways involving delayed gastric emptying and modulation of gut hormone secretion (e.g., GLP-1, PYY, and ghrelin) [2].

Dairy products have been investigated in relation to appetite due to their protein content [8]. Interestingly, some dairy products such as cow cheese have shown differences in satiety according to their nutritional composition. Hansen et al. [9] observed a lower total energy consumption and a higher satiety effect with high-protein/low-fat cheese (HP/LF) consumption than with high-protein/high-fat (HP/HF) and low-protein/high-fat (LP/HF) cheeses, suggesting that this type of cheese could increase the sensation of satiety and lower caloric consumption, regardless of its fat content.

Goat milk and its derivatives have gained attention due to their nutritional properties, which include a higher content of short- and medium-chain fatty acids and better digestibility in comparison with cow’s dairy [10]. In addition, it has been reported that goat dairy could have a slightly higher satiety effect than cow’s dairy [11]. Additionally, Santurino et al. [12] reported that during a 12-week clinical trial, daily consumption of 60 g of goat cheese enriched with *n*-3 polyunsaturated fatty acids (PUFA) and conjugated linoleic acid (CLA) significantly improved lipid profiles (increased high-density lipoprotein (HDL)) and reduced *C*-reactive protein in overweight adults with cardiovascular risk. These provide evidence for dietetic recommendations based on nutritional personalization, especially in populations with metabolic disorder risks, guiding the development of dairy functional products with specific benefits. However, as with other animal-derived foods, fat content remains an important factor in determining the effect of its consumption. Delgadillo-Puga et al. [13] studied different goat cheeses marketed in Mexico with a focus on their fat content, fatty acid composition and their inferred effect on specific indices such as cholesterol/saturated-fat, atherogenicity, thrombogenicity and health-promoting indices; they found that goat cheese fat can both positively and negatively affect consumer health.

As the incorporation of goat cheese in the diet could represent an innovative strategy for the improvement of the satiety sensation, which is a promising strategy for supporting weight management interventions, this study aims to evaluate the satiety effect, changes in acylated ghrelin concentrations, and anthropometric outcomes in volunteers under a dietary intervention, which includes two commercial goat cheese consumptions within an energy-restricted trial. The present pilot trial hypothesized that the nutritional profiles of the two commercial goat cheeses would influence postprandial subjective appetite ratings or acylated ghrelin suppression, and that individual variability in sustained satiety at 120 min would be significantly associated with short-term body weight reduction.

## 2. Materials and Methods

### 2.1. Study Approval

**Figure 1:**
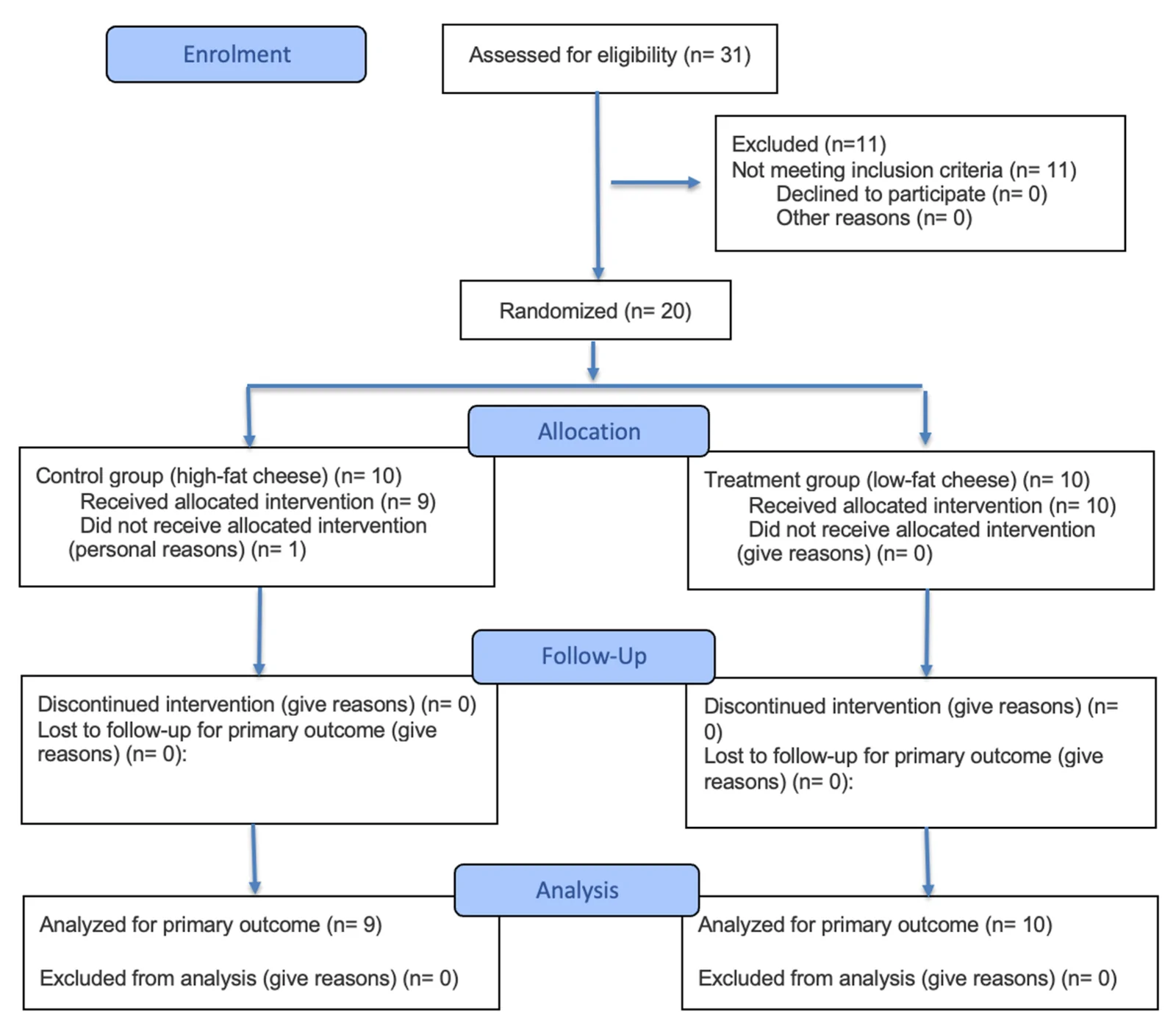
CONSORT 2025 Flow Diagram of the progress through the phases of a randomized trial involving two groups (including enrolment, intervention allocation, follow-up, and data analysis).

This study was a randomized, controlled, blinded, two-arm parallel-group clinical trial carried out according to the Consolidated Standards of Reporting Trials (CONSORT) guidelines (Figure 1). Ethical approval for the study was obtained from the Bioethics Committee of the Facultad de Medicina de la Universidad de Colima (2025-8b) on 6 May 2025. The written informed consent form was distributed to all participants and signed by each volunteer prior to their inclusion in the trial. All subjects provided written, informed consent. The present clinical trial was registered as NUTRICHEESE: RPCEC00000462 in the Cuban Public Registry of Clinical Trials (RPCEC) Database (https://trialsearch.who.int/Trial2.aspx?TrialID=RPCEC00000462, accessed on 14 September 2026).

### 2.2. Volunteers

Thirty-one volunteers were screened to determine their eligibility for the trial, and all were recruited at the Centro de Biotecnología Genómica—Instituto Politécnico Nacional, located in Reynosa, Tamaulipas, Mexico. The inclusion criteria required men or women aged between 18 and 55 with a body mass index (BMI) ≥ 25 kg/m^2^ who were motivated to improve their health by losing weight and changing their eating habits. All the volunteers reported being frequent cheese consumers (eating cheese one to three times per week). During the screening interview, volunteers were also asked about their habitual dairy consumption. On average, volunteers reported consuming dairy products five days per week, with an average intake of 1.47 servings per day on days of consumption. None of the enrolled participants reported following a vegetarian dietary pattern, and all of them were willing to pause their consumption of milk, dairy products, probiotics, fermented foods, and cheese not provided by the clinical trial, as well as other food supplements. The exclusion criteria were individuals with type 1 or 2 diabetes, hypertension, chronic or acute diseases at the time of the interview, alcoholism or drug addiction, those who smoked (>10 cigarettes a day), were pregnant or breastfeeding, or who used medical treatments or dietary supplements as evaluated by a self-reported form, had allergies and/or lactose intolerance, or practiced sports or strenuous exercise. Volunteers deemed unlikely to comply with the study protocol were excluded. Recruitment and follow-up periods were conducted between March 2026 and June 2026.

### 2.3. Study Design

The study was conducted as a randomized controlled trial with blinded outcome assessment and statistical analysis, where the sample collectors and researchers were not aware of the assigned group. Following the initial screening, twelve volunteers were excluded because they did not meet the inclusion criteria. The sample size calculation was based on the results described by Seyssel et al. [14]. Assuming a significance level α = 0.05 and a statistical power (1 − β = 0.80), initial estimations were conducted in G*Power 3.1.9.7. Although the software suggested a minimal cohort size, we recruited 10 volunteers per group. This enrollment was set to ensure sufficient statistical power for the multi-way fixed effects (group × week × time) within our generalized estimating equations (GEE) framework, while accounting for an anticipated attrition rate of up to 20% over the 4-week caloric restriction protocol. The 20 volunteers were randomly assigned to either the treatment group (n = 10; LF cheese) or the control group (n = 10; HF cheese) for dietary intervention.

Randomization was performed using the Statistical Analysis System software, version 9.2 (SAS Inst. Inc., Cary, NC, USA). The researchers who generated the randomization were completely uninvolved in volunteer recruitment or any other direct interactions with the study volunteers.

### 2.4. Dietary Assessment

At the beginning of the intervention, volunteers received a 7-day menu outlining specific meal frequencies and portion sizes for fruits, vegetables, cereals, etc. The meal plan included goat cheese consumption (80 g per day, three days a week, over four weeks). The menus were adjusted according to the volunteers’ energy requirements in a state of caloric restriction (20% average deficit). To homogenize the diets, the energy requirement of each volunteer was averaged, and 1200, 1260, 1320, 1440, 1500, 1560, 1620, and 1740 kilocalorie diets were provided with a ratio of 50% carbohydrates, 20% proteins, and 30% fat. The menu included chicken, beef, fish, eggs, turkey ham, beans, peas, lentils, chickpeas, avocados, nuts, oats, bread, and different kinds of vegetables.

Over the course of the clinical trial, each volunteer consumed twelve breakfasts. These meals consisted of 80 g of cheese (from their assigned group), one serving of cereals, and one serving of vegetables. A nutritionist prepared all breakfasts in accordance with standard sanitary protocols, using separate cooking pans for the two cheese variants without any additional animal or vegetable fats. Breakfast was provided to the volunteers in a dining area of the Centro de Biotecnología Genómica to verify the total consumption of the cheese provided, at a set time between 8:30 and 10:30 a.m., after a 10 to 12 h overnight fast. Adherence to the study protocol was monitored through regular in-person and telephone check-ins. Additionally, volunteers had open communication with the nutritionist to resolve doubts via text message, email, or in person.

Volunteers were instructed to avoid ingestion of milk, yogurt, cream, probiotics, and other cheeses, and to consume only what was recommended in the diet, such as legumes, nuts, oats, bread, chicken, beef, fish, eggs, chicken or turkey ham or sausage, and different types of vegetables and fruits. Exercise and moderate or intense physical activities were restricted to avoid generating additional caloric expenditure.

Recruitment and follow-up periods were conducted between March 2026 and June 2026.

### 2.5. Cheese Processing

Individual packages of commercially available goat cheese were acquired every week of the clinical trial. A supermarket conventional cheese (SCC) was purchased at a local supermarket in Reynosa, Tamaulipas, Mexico, while an artisanal regional cheese (ARC) was purchased from a small local entrepreneur located in Monterrey, Nuevo Leon, Mexico. We selected these two specific goat cheese variants to test some of the commercial food choices that consumers buy in the local market. The SCC variant represents a conventional, mass-distributed product from regional supermarkets. The ARC variant represents a traditional fresh cheese sold through local dairy outlets via a short regional supply chain. Before intervention, the nutritional content of both cheeses used in the dietary intervention was evaluated in triplicate from random samples of prepared batches of cheese. The caloric content of the cheeses was 189 kcal vs. 231 kcal per 80 g portion for the ARC and SCC cheeses, respectively. The chemical composition was determined according to Mexican standards, which are mainly based on the methods of the Association of Official Analytical Chemists [15].

### 2.6. Subjective Appetite and Combined Satiety Index (CSI)

**Figure 2:**
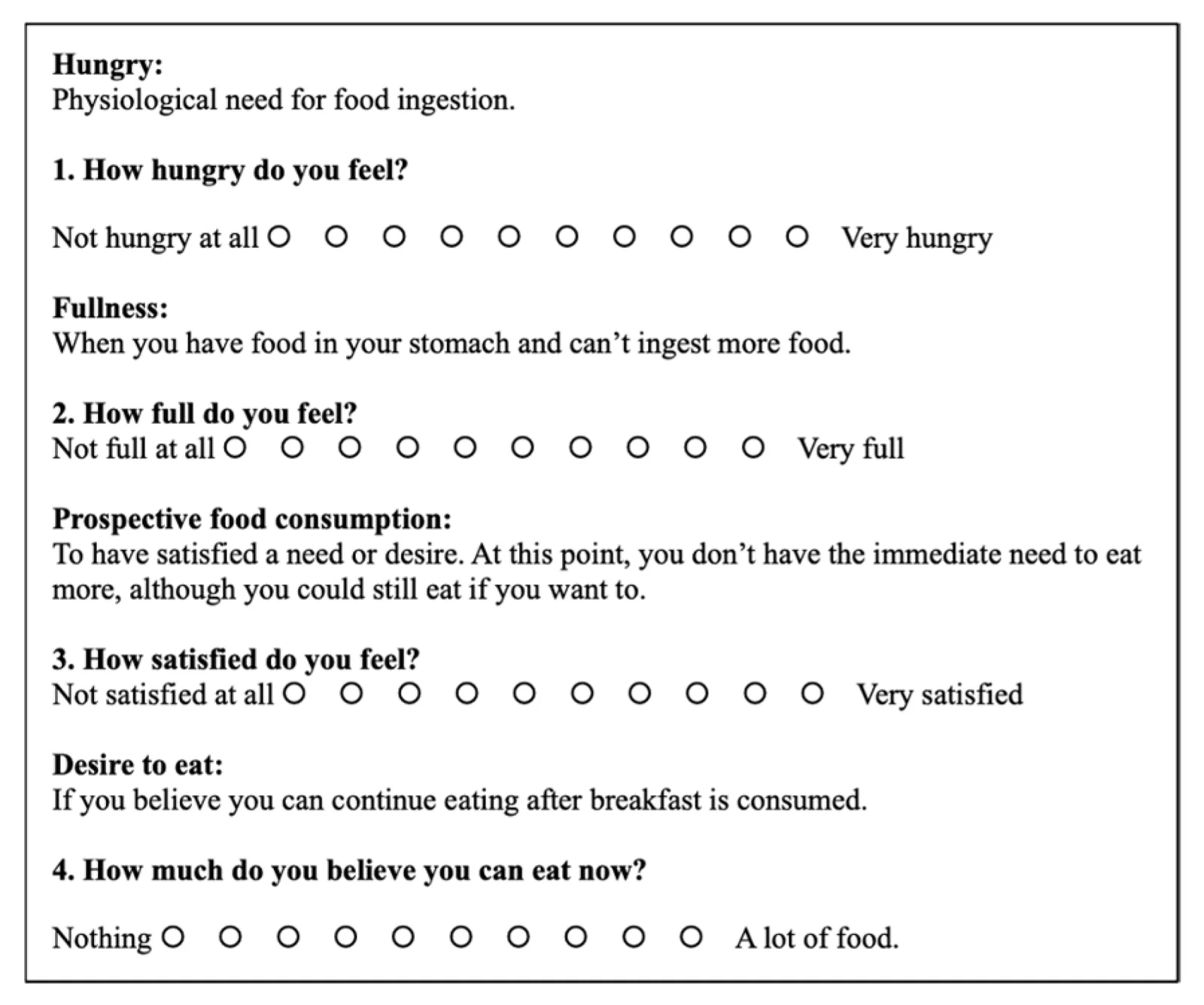
Visual Analogue Scale (VAS) applied to volunteers. Response options consisted of words anchored at the beginning and end, describing the extremes. To determine the subjective profile of hunger, fullness, prospective food consumption, and desire to eat, responses were rated on 10-point visual analogue scales (VAS), each point representing one centimeter, anchored with “not at all” and “very/a lot.” The scales were anchored only at the absolute extremes to allow for a continuous biometric measurement. Each question was preceded by a brief description defining the appetite sensation being assessed.

The primary outcome was the postprandial subjective satiety response, assessed using the Combined Satiety Index (CSI). The CSI was selected as the primary efficacy endpoint because it integrates multiple complementary dimensions of appetite into a single validated composite measure, providing a more comprehensive and robust assessment of postprandial satiety than any individual visual analogue scale (VAS) [9]. To determine the sensory satiety profile, a 10-point visual analog scale (VAS) was used to assess hunger, desire to eat, fullness, and prospective food consumption (Figure 2), and the Combined Satiety Index (CSI) was then calculated. VAS has been previously validated and can be administered digitally or on paper [16,17]. In our study, the VAS was digitized through Google Forms, and the link to access it was shared via mobile phone. This scale was required on the three days of the week during which the assigned breakfast was consumed. During the four weeks of the intervention, volunteers completed the VAS at times T0, T30, and T120 after breakfast, all times when the sensation of satiety can be evaluated, making it possible to assess the intensity of the different appetite ratings [18].

Subjective appetite was evaluated under fasting conditions (0 min) and at 30 and 120 min after breakfast during each of the four weekly intervention appointments. The primary efficacy endpoint was the longitudinal difference in postprandial CSI between volunteers consuming the two goat cheese options.

A Combined Satiety Index (CSI) was calculated using the following formula [11]:

CSI = [Fullness + (10 − Desire to eat) + (10 − Hungry) + (10 − prospective food consumption)]/4(1)

CSI values fall within the range of 1 and 10, with 1 being the maximum appetite sensation and 10 the minimum appetite sensation [11].

Secondary efficacy outcomes included individual appetite sensations (hunger, fullness, desire to eat, and prospective food consumption), postprandial acylated ghrelin concentrations, and changes in anthropometric variables, including body weight, body mass index (BMI), waist circumference, hip circumference, and waist-to-hip ratio.

To characterize the evolution of appetite throughout the intervention, sustained postprandial satiety was additionally evaluated as the change in CSI between fasting and 120 min after breakfast (ΔCSI_0–120_). Finally, a prespecified exploratory analysis investigated whether sustained postprandial satiety, quantified as the volunteer-specific mean CSI measured 120 min after breakfast across the intervention, was cross-sectionally associated with body weight reduction.

### 2.7. Postprandial Acylated Ghrelin Concentrations

At the beginning and at the end of the dietary intervention, a peripheral venous catheter was placed by trained personnel in each volunteer (which was maintained for approximately 2.5 h) for the collection of three blood samples before breakfast (T0) and at 30 and 120 min (T30 and T120) after breakfast. During this time, the volunteers were instructed not to consume any food or beverage. Acylated ghrelin was quantified at T0 before breakfast and at T30 after breakfast using the commercial kit Human A-GHRL (Acylated Ghrelin) ELISA Kit (E-EL-H2002) from Elabscience^®^ (Houston, TX, USA) at the beginning and end of the intervention.

### 2.8. Anthropometric and Body Composition Variables

Body weight was measured using a digital scale (OMRON, HBF-514C-LA, Muko, Kyoto, Japan) while volunteers wore minimal clothing and no shoes. Body mass index (BMI) was calculated as body mass (kg) divided by height in meters squared (kg/m^2^). Waist, hip, and abdominal circumferences (cm) were measured according to standard procedures [19] using an anthropometric tape (Lufkin W606PM, Apex Tool Group, Sparks, MD, USA). The waist–hip ratio (WHR) was calculated as the waist circumference divided by the hip circumference. To minimize measurement variation, all evaluations were performed by the same anthropometrist.

### 2.9. Statistical Analysis

Continuous variables are presented as mean ± standard deviation (SD) or as estimated marginal means (EMMs) with corresponding 95% confidence intervals (95% CI). Categorical variables are presented as frequencies and percentages. Baseline characteristics were compared using Welch’s *t*-test or Fisher’s exact test.

Because repeated observations were obtained from each volunteer, all efficacy analyses were performed using longitudinal models. The primary outcome (Combined Satiety Index), individual appetite sensations, and ΔCSI_0–120_ were analyzed using generalized estimating equations (GEE) with an exchangeable working correlation structure. This approach was selected to account for within-subject correlation across repeated measurements while retaining the individual appetite-related outcomes in their original, clinically interpretable scales, rather than reducing them to exploratory composite dimensions. We selected the mean CSI at 120 min because it reflects sustained postprandial fullness just before the subsequent meal window. While ΔCSI_0–120_ captures the magnitude of change from fasting, the absolute CSI score at 120 min represents the actual, net satiety state of the individual at the end of the postprandial observation period. Fixed effects included treatment group, intervention week, and postprandial time, together with the corresponding interaction terms, assessed using Wald tests. Postprandial acylated ghrelin concentrations were analyzed using analogous GEE models after being natural logarithm transformed. Changes in anthropometric outcomes were evaluated using analysis of covariance (ANCOVA), with the corresponding baseline value included as a covariate.

The association between sustained postprandial satiety and body weight reduction was investigated using multivariable linear regression, with baseline BMI included as an adjustment covariate. To improve the robustness of statistical inference given the relatively small sample size, regression coefficients and confidence intervals were internally validated using a bias-corrected and accelerated (BCa) bootstrap procedure based on 10,000 volunteer-level resamples and heteroscedasticity-consistent HC3 standard errors. Model performance was summarized using both R^2^ and adjusted R^2^. No adjustment for multiple comparisons was applied.

All statistical analyses were performed using IBM SPSS Statistics version 30 and R version 4.4.1. All tests were two-sided, and statistical significance was defined as *p* < 0.05.

## 3. Results

### 3.1. Study Population and Baseline Characteristics

A total of 19 volunteers completed the four-week dietary intervention and were included in the final longitudinal analyses: nine volunteers in the high-fat goat cheese group (HF) and ten in the reduced-fat goat cheese group (LF). One volunteer in the HF group dropped out due to personal reasons.

Baseline demographic and anthropometric characteristics were balanced between the randomized intervention groups (Table 1). Volunteers had a mean age of 32.89 ± 10.23 years in the HF group and 34.30 ± 11.70 years in the LF group (*p* = 0.783). Mean baseline body mass index (BMI) was 30.86 ± 3.60 kg/m^2^ and 32.09 ± 6.24 kg/m^2^, respectively (*p* = 0.601), with BMI values ranging from 25.8–35.8 and 25.3–43.7 kg/m^2^, respectively. Baseline body weight, waist circumference, hip circumference, and waist-to-hip ratio were also broadly similar between groups (Table 1).

### 3.2. Nutritional Characterization of the Tested Commercial Goat Cheeses

The nutritional composition of the cheeses used in the dietary intervention is shown in Table 2. No significant differences were observed in the nutritional components analyzed except for fat content (23.32 ± 0.71 vs. 17.28 ± 1.51%, *p* = 0.002) and ash content (1.46 ± 0.00 vs. 3.02 ± 0.18%, *p* < 0.001) in the SCC and ARC cheeses, respectively, per 100 g. The measured fat variance between formulations reflects the natural composition of commercial products currently available to consumers in the marketplace, rather than a laboratory-controlled formulation in compliance with a specific reduced-fat regulated labeling category. While acknowledging their distinct commercial features, these cheeses were hereinafter designated as high-fat (HF; corresponding to SCC) and low-fat (LF; corresponding to ARC) cheeses, based on their significant difference in total lipid concentration, which represents a primary dietary bioactive component strongly involved in postprandial satiety.

The 80 g cheese portion represented a meaningful component of the volunteers’ daily dietary intake. The LF and HF cheeses provided 189.5 and 231.5 kcal per portion, respectively, corresponding to 15.8% and 19.3% of the 1200 kcal diet and 10.9% and 13.3% of the 1740 kcal diet.

### 3.3. Postprandial Subjective Satiety Response

The primary outcome was evaluated through the longitudinal postprandial subjective satiety response using the Combined Satiety Index (CSI). Across the four-week intervention, both goat cheese options elicited a marked increase in subjective satiety after breakfast. As shown in Table 3, mean CSI values increased from 3.57 at fasting to 5.91 at 30 min in the HF group, and from 3.64 to 6.29 in the LF group. Perceived satiety remained consistently above fasting levels at 120 min in both groups, reaching estimated mean CSI values of 5.19 and 5.52, respectively.

The magnitudes of both the acute and sustained satiety responses were highly comparable across cheese formulations. The adjusted between-group difference in the change from fasting to 30 min was 0.31 points (95% CI: −0.67 to 1.30; *p* = 0.533), whereas the adjusted difference from fasting to 120 min was 0.27 points (95% CI: −0.79 to 1.33; *p* = 0.621). Longitudinal GEE analysis confirmed no statistical evidence that the overall CSI pattern differed according to cheese selection (global group-related postprandial interaction, *p* = 0.601). Furthermore, no evidence indicated that the between-group pattern changed across the intervention weeks (group × week × time interaction, *p* = 0.427).

Individual subjective appetite sensations consistently supported the primary outcome. In both groups, hunger and prospective food consumption ratings decreased substantially after breakfast, while fullness and satiety-related desire-to-eat scores increased, showing peak effects at 30 min that remained evident at 120 min. Longitudinal GEE modeling revealed no significant between-group differences in the fasting-to-30 min change for hunger (*p* = 0.653), fullness (*p* = 0.847), satiety-related desire-to-eat (*p* = 0.952), or prospective food consumption (*p* = 0.642). Similarly, individual appetite patterns from fasting to 120 min did not differ significantly between the cheese groups (*p* > 0.05 for all domains), confirming that the nutritional profile of the cheese did not alter subjective appetite patterns (Table 4).

### 3.4. Acylated Ghrelin Response

Plasma concentrations of acylated ghrelin exhibited high interindividual variability. After natural logarithm transformation and longitudinal GEE modeling, no significant differences were observed between the cheeses regarding the postprandial ghrelin response. During week 1, the ratio comparing the postprandial T30/T0 response in the LF group relative to the HF group was 1.12 (95% CI: 0.52–2.40; *p* = 0.771). During week 4, the corresponding response ratio was 0.80 (95% CI: 0.33–1.98; *p* = 0.634). The group × week × time interaction was also non-significant (ratio: 0.72; 95% CI: 0.28–1.85; *p* = 0.492), showing that hormonal suppression did not diverge between cheese profiles over the course of the intervention (Table 5).

### 3.5. Anthropometric Outcomes and Weight Loss

Both intervention groups experienced reductions in body weight and anthropometric measurements during the four-week hypocaloric intervention. Mean weight loss was −2.49 ± 1.31 kg in the HF group and −2.24 ± 1.50 kg in the LF group. After ANCOVA adjustment for baseline body weight, the estimated final between-group difference was 0.38 kg (95% CI: −0.92 to 1.69; *p* = 0.566). No significant differences were observed for BMI (adjusted difference: −0.04 kg/m^2^; *p* = 0.846), waist circumference (adjusted difference: 0.57 cm; *p* = 0.669), hip circumference (adjusted difference: −0.13 cm; *p* = 0.863), or waist-to-hip ratio (adjusted difference: 0.004; *p* = 0.777) (Table 6). These findings indicate that the anthropometric response was predominantly driven by the energy-restricted dietary regimen rather than modified by the cheese fat content.

### 3.6. Evolution of Sustained Satiety and Association with Weight Loss

The longitudinal evolution of sustained satiety, evaluated as the change in CSI from fasting to 120 min (ΔCSI_0–120_), increased modestly over time (Table 7). Each additional intervention week was significantly associated with an estimated 0.164-point increase in ΔCSI_0–120_ (β = 0.164; 95% CI: 0.058 to 0.270; *p* = 0.003). This temporal improvement occurred similarly in both groups, as neither the main effect of cheese type (*p* = 0.810) nor the group × week interaction (*p* = 0.234) reached statistical significance. Complementarily, the cumulative postprandial satiety response (CSI AUC_0–120_) was comparable between the HF and LF groups (641.8 ± 129.3 vs. 680.1 ± 62.9 units·min, respectively; *p* = 0.437).

A prespecified multivariable linear regression analysis was performed to determine whether individual sustained satiety predicted weight loss independently of cheese assignment. The volunteer-specific mean CSI measured at 120 min after breakfast, averaged across the intervention weeks, was positively associated with weight reduction after adjustment for baseline BMI. As shown in Table 8, each one-point higher sustained CSI score was significantly associated with an additional 0.476 kg of body weight loss (β = 0.476 kg; 95% BCa CI: 0.057 to 0.933; bootstrap *p* = 0.033). Conversely, baseline BMI was independently associated with smaller weight reductions (β = −0.172 kg per kg/m^2^; 95% BCa CI: −0.258 to −0.086; bootstrap *p* = 0.004). Overall, the multivariable model explained 50.8% of the observed variability in weight loss (R^2^ = 0.508; adjusted R^2^ = 0.447).

The multivariable model explained 50.8% of the observed variability in weight loss (R^2^ = 0.508; adjusted R^2^ = 0.447), which represents a meaningful explanatory capacity in clinical settings given that only two independent predictors were evaluated.

## 4. Discussion

Here we evaluated the postprandial satiating effect of consuming two commercially available goat cheeses differing in fat content (high-fat versus low-fat) in volunteers undergoing a calorie-restricted dietary intervention. As expected, at the end of the trial, caloric restriction induced significant intra-group reductions in body weight and BMI across both groups. However, our findings demonstrated that differences in fat content, particularly in the goat cheeses tested, did not have a significant effect on changes in the volunteers’ body composition between the cheese groups after four weeks of calorie restriction. These results suggest that regular consumption of goat cheese, regardless of its fat profile, can be safely included in a weight management context.

The effect of consuming cheese with different fat contents has been reported mainly in studies conducted with cow’s milk, and the current consensus remains highly controversial. Raziani et al. [20] previously found that weight loss after consuming low-fat cheese was numerically less than after consuming regular cheese, although there were no statistically significant differences between them. Conversely, Pedersen and Kirkhus [21] challenged those results and demonstrated with prediction equations that fat content does have an important biological effect on lipid profiles, remarking that dietary studies with small sample sizes can lead to inadequate or overgeneralized conclusions regarding the effect of saturated fat on consumers’ health.

In our study, the lack of significant anthropometric differences between the HF and LF groups indicates that when overall energy deficit is controlled, the moderate cheese fats do not alter the downward trend of body weight. However, these results should be confirmed since, although dietary counseling and weekly check-ins were performed, exact daily caloric intake and overall dietary compliance were not quantitatively assessed using weighted food records or daily food records. Therefore, potential differences in adherence to the prescribed hypocaloric diets between volunteers or groups cannot be excluded and may have contributed to the observed anthropometric outcomes.

Satiety is considered a fundamental factor in stimulating adherence to long-term dietary programs. From a nutritional perspective, dietary fats are considered potent regulators of satiety signals due to their ability to delay gastric emptying [22,23]. Fat present in foods stimulates the secretion of cholecystokinin (CCK) in the duodenum, which inhibits antral gastric motility and increases pyloric tone [24]. However, our longitudinal GEE analysis showed no evidence that the overall Combined Satiety Index (CSI) pattern differed according to cheese type (*p* = 0.601). Nowadays, appetite regulation models extend beyond individual mechanisms such as gastric emptying or CCK secretion and instead emphasize the dynamic integration of gastrointestinal, vagal, endocrine, metabolic, and central neural signals [25,26]. Nutrient sensing in the gastrointestinal tract promotes the release of several appetite-regulating peptides, including CCK, GLP-1, PYY, and ghrelin, which interact with vagal pathways and hypothalamic and brainstem circuits involved in the regulation of food intake [25,26]. Moreover, these short-term meal-related signals interact with longer-term metabolic and adiposity-related signals, including insulin and leptin, supporting the concept that postprandial satiety emerges from an integrated gut–brain and metabolic network rather than from the action of a single hormone or nutrient-related pathway [26]. Because no statistically significant differences in postprandial satiety were detected between the assigned cheese groups and considering that the low-fat option provided a lower energy density per portion (approximately 42 kcal less per 80 g portion, representing an 18.1% reduction in energy content), this commercial variant could offer a practical caloric advantage for dietary planning. This formulation may facilitate adherence to energy-restricted regimens without triggering the dietary anxiety frequently associated with reduced portion sizes. Nevertheless, longer-term clinical trials with larger sample sizes are required to confirm this potential benefit. Furthermore, studies characterizing the specific structural and rheological properties of these commercial goat cheese matrices will be essential to define their precise role in appetite regulation. These considerations are consistent with Rubio-Martín et al. [11], who also observed high satiety profiles following goat dairy consumption, potentially driven by the superior digestibility and distinct medium-chain fatty acid profile inherent to caprine milk matrices.

Our exploratory multivariable regression model revealed that individual biological variability in sustained postprandial satiety at 120 min was significantly associated with short-term body weight reduction, suggesting a concurrent relationship between satiety perceptions and weight management success (β = 0.476 kg per 1-point CSI increase; bootstrap *p* = 0.033). While the breakfasts were tightly standardized and monitored to verify total consumption, substantial interindividual variability was observed in CSI and individual appetite sensations (hunger, fullness, and desire to eat). Such variability is consistent with the current view of obesity, a condition characterized by differences in metabolic phenotypes and responses to dietary exposures [27,28]. The data showed that the consumption of standardized breakfasts containing goat cheese was associated with a postprandial decrease in plasma concentrations of acylated ghrelin; however, it is important to note that the observed interindividual variability in subjective satiety cannot be explained by ghrelin dynamics alone. Postprandial appetite responses reflect the integration of gastrointestinal peptides, adiposity-related signals such as leptin, insulin sensitivity, inflammatory status, gastric signaling, and central responsiveness to peripheral metabolic signals, rather than the isolated action of a single appetite-regulating hormone [25,27]. This biological heterogeneity also includes the genetic background and the individual biological responses to dietary exposures and interventions [27,28]. Although the changes varied descriptively across cheese types and over the weeks, no significant differences were observed between groups at the evaluated time points; however, the lack of statistical significance in ghrelin suppression between groups should be interpreted cautiously due to a lack of statistical power rather than assuming an absence of physiological effect. The literature reports that postprandial ghrelin responses often show highly contrasting results in individuals with obesity, particularly after consuming high-fat foods [29,30].

An important point to address is that baseline acylated ghrelin concentrations decreased by the fourth week of intervention in most of our comparisons. Previously, Hansen et al. [31] reported a significant 12% increase in baseline ghrelin concentrations after weight loss, which positively correlated with weight reduction, providing evidence of a compensatory hormonal drive to regain weight. Our contrasting decrease in fasting ghrelin can be explained by the specific nature of the continuous caloric restriction without exhaustive exercise to which the volunteers were subjected, which coincides with the findings of Malin et al. [32]. The observed decrease in baseline acylated ghrelin at week 4 represents a descriptive trend under continuous caloric restriction; however, as previously stated, the acylated ghrelin response observed in the present study should be interpreted as one component of a broader integrated appetite-regulatory system rather than as a complete mechanistic explanation for the subjective satiety response. Despite using visual analogue scales (VAS), which represent the most widely used and validated method for assessing subjective appetite sensations, it is important to consider that VAS captures subjective perceptions that may differ from objective measurements of subsequent food consumption [33]. Due to the limited sample size (n = 19), this study should be considered a pilot study. While the inferential statistics for several sub-metrics were non-significant, the robust HC3 standard errors and 10,000 bias-corrected and accelerated (BCa) bootstrap resamples internally validated the regression coefficients, supporting the reliability of the main findings. However, several methodological points should be considered when interpreting the findings of this study, which concurrently offer valuable pathways for future confirmatory research. First, while utilizing commercially available cheeses provides a highly practical comparison representative of options currently available to consumers in the marketplace, this approach inherently limited the isolation of fat content as a single variable, given that the matrices were manufactured by different producers and varied in ash content. Future studies characterizing the precise physical and rheological properties of these matrices will be essential to define their distinct roles in appetite regulation. Second, although individualized hypocaloric diets were prescribed and adherence was qualitatively monitored, actual energy and nutrient intake and overall dietary adherence were not quantitatively assessed during the intervention. Consequently, potential differences in adherence to the prescribed diets cannot be excluded as contributors to the observed anthropometric changes, limiting our ability to attribute these outcomes specifically to the tested cheese products. Future trials should incorporate quantitative dietary assessment throughout the intervention to better account for dietary adherence. Third, while our exploratory findings highlight marked interindividual variations in satiety, it is important that future large-scale trials include additional endocrine, inflammatory, or metabolic markers in order to comprehensively map these heterogeneous appetite responses among individuals with overweight and obesity. Finally, because this pilot evaluation did not include a non-cheese reference meal, the observed postprandial responses reflect the comparable satiety profiles between the two tested variants under energy restriction, rather than establishing whether goat cheese intrinsically enhances satiety relative to other dietary choices. Additionally, true volunteer blinding could not be confirmed due to organic sensory and texture variations between the commercial products, and a formal allocation-guessing assessment was not performed. These considerations highlight this study as a pilot investigation, outlining the need for future large-scale, long-term clinical trials to confirm these positive neuroendocrine dynamics and their long-term impact on dietary adherence during weight loss.

## 5. Conclusions

Continuous calorie restriction drives overall weight loss and anthropometric improvements independently of the specific goat cheese consumed. In this study, which evaluated two distinct commercially available goat cheese products, the variant with a lower fat concentration and lower energy density produced a statistically nonsignificant difference in postprandial subjective satiety compared with the conventional high-fat product. These findings suggest that individuals managing postprandial appetite have the choice to use practical, commercially available local food products instead of highly industrialized dietary products. Future large-scale clinical trials must evaluate these long-term neuroendocrine dynamics over extended periods to confirm their true impact on dietary adherence.

## Figures and Tables

**Table 1:** Baseline characteristics of volunteers according to intervention group.

Characteristic	High-Fat Goat Cheese (n = 9)	Low-Fat Goat Cheese (n = 10)	*p*-Value
Age (years)	32.9 ± 10.2	34.3 ± 11.7	0.783
Female, n (%)	5 (55.6)	7 (70.0)	0.650 ^1^
Body weight (kg)	84.1 ± 14.6	80.9 ± 13.6	0.630
Body mass index (kg/m^2^)	30.9 ± 3.6	32.1 ± 6.2	0.601
Waist circumference (cm)	94.9 ± 10.7	93.6 ± 12.7	0.813
Hip circumference (cm)	111.9 ± 8.9	110.8 ± 13.5	0.837
Waist-to-hip ratio	0.85 ± 0.08	0.85 ± 0.06	0.929
Fasting glucose (mg/dL)	91.6 ± 7.6	96.3 ± 12.2	0.321

Continuous variables are presented as mean ± standard deviation and were compared using Welch’s *t*-test. ^1^ Sex was compared using Fisher’s exact test.

**Table 2:** Nutritional content in 100 g of the cheeses used for the dietary intervention *.

Nutriment	HF Cheese	LF Cheese	*p*-Value ^†^
Carbohydrates, %	3.18 ± 0.47	3.41 ±1.68	0.860
Protein, %	16.70 ± 1.04	16.94 ± 1.38	0.829
Fat, %	23.32 ± 0.71	17.28 ± 1.51	0.002
Ashes, %	1.46 ± 0.00	3.02 ± 0.18	<0.001
Fiber, %	0.10 ± 0.00	0.10 ± 0.00	0.267
Humidity, %	55.35 ± 1.27	59.34 ± 2.46	0.078

* Values are means and standard deviations. HF: High-fat; LF: Low-fat. ^†^: Student’s *t*-test for independent samples.

**Table 3:** Longitudinal analysis of the Combined Satiety Index.

Outcome	High-Fat Cheese	Low-Fat Cheese	Adjusted Difference, LF-HF	95% CI	*p*-Value
CSI at fasting	3.57	3.64	-	-	-
CSI at 30 min	5.91	6.29	-	-	-
CSI at 120 min	5.19	5.52	-	-	-
Change, 0–30 min	+2.34	+2.65	0.31	−0.67 to 1.30	0.533
Change, 0–120 min	+1.62	+1.89	0.27	−0.79 to 1.33	0.621
Global group-related postprandial interaction	-	-	-	-	0.601
Group × week × time interaction	-	-	-	-	0.427

Values at fasting, 30 min, and 120 min are estimated marginal means averaged across the four intervention weeks. Adjusted between-group differences and interaction *p*-values were derived from generalized estimating equations (GEE) with an exchangeable working correlation structure, using Wald tests. CSI, Combined Satiety Index; CI, confidence interval; HF, high-fat goat cheese; LF, low-fat goat cheese.

**Table 4:** Subjective appetite responses at 30 and 120 min.

**Outcome**	**HF Fasting**	**HF 30 min**	**LF Fasting**	**LF 30 min**	**LF-HF Difference in Change, 0–30 min (95% CI)**	***p*-Value**
Hunger	7.71	2.86	7.38	2.07	−0.46 (−2.45 to 1.53)	0.653
Fullness	2.24	7.50	2.14	7.62	0.22 (−1.97 to 2.40)	0.847
Satiety-related desire-to-eat score	2.06	7.85	2.17	7.88	−0.07 (−2.34 to 2.20)	0.952
Prospective food consumption	8.17	3.15	8.06	2.52	−0.51 (−2.69 to 1.66)	0.642
**Outcome**	**HF Fasting**	**HF 120 min**	**LF Fasting**	**LF 120 min**	**LF-HF Difference in Change, 0–120 min (95% CI)**	***p*-Value**
Hunger	7.71	4.19	7.38	3.64	−0.21 (−2.34 to 1.91)	0.843
Fullness	2.24	6.24	2.14	6.25	0.11 (−1.99 to 2.20)	0.919
Satiety-related desire-to-eat score	2.06	6.58	2.17	6.59	−0.09 (−2.17 to 1.98)	0.930
Prospective food consumption	8.17	4.69	8.06	3.93	−0.65 (−2.82 to 1.51)	0.555

Values at fasting, 30 min, and 120 min are estimated marginal means averaged across the four intervention weeks. HF, high-fat goat cheese; LF, low-fat goat cheese; CI, confidence interval.

**Table 5:** Acylated ghrelin response according to intervention group.

Contrast	Ratio of Postprandial Responses, LF vs. HF	95% CI	*p*-Value
Week 1, T30/T0 response	1.12	0.52–2.40	0.771
Week 4, T30/T0 response	0.80	0.33–1.98	0.634
Group × week × time interaction	0.72	0.28–1.85	0.492

Values are exponentiated estimates from generalized estimating equations and represent ratios of the postprandial T30/T0 response in the low-fat group relative to the high-fat group. A ratio of 1 indicates an equivalent response. LF, low-fat goat cheese; HF, high-fat goat cheese; CI, confidence interval.

**Table 6:** Changes in anthropometric outcomes after four weeks.

Outcome	HF Change, Mean ± SD	LF Change, Mean ± SD	Adjusted Difference, LF-HF	95% CI	*p*-Value
Body weight, kg	−2.49 ± 1.31	−2.24 ± 1.50	0.38	−0.92 to 1.69	0.566
BMI, kg/m^2^	−0.91 ± 0.50	−0.88 ± 0.56	−0.04	−0.49 to 0.40	0.846
Waist circumference, cm	−3.11 ± 1.32	−2.60 ± 3.07	0.57	−2.06 to 3.21	0.669
Hip circumference, cm	−2.61 ± 1.11	−2.75 ± 1.55	−0.13	−1.55 to 1.30	0.863
Waist-to-hip ratio	−0.009 ± 0.016	−0.005 ± 0.034	0.004	−0.021 to 0.028	0.777

Changes were calculated as the final value minus the baseline value. HF, high-fat goat cheese; LF, low-fat goat cheese; SD, standard deviation; CI, confidence interval; BMI, body mass index.

**Table 7:** Longitudinal evolution of sustained satiety and cumulative satiety response.

Analysis	Estimate	95% CI	*p*-Value
Weekly change in ΔCSI_0–120_	0.164	0.058 to 0.270	0.003
Main effect of cheese type	-	-	0.810
Group × week interaction	-	-	0.234
CSI AUC_0–120_ (HF), units·min	641.8 ± 129.3	-	-
CSI AUC_0–120_ (LF), units·min	680.1 ± 62.9	-	0.437

ΔCSI_0–120_ represents the change in the Combined Satiety Index between fasting and 120 min after breakfast. Values are presented as estimated regression coefficients or mean ± standard deviation, as appropriate. HF, high-fat goat cheese; LF, reduced-fat goat cheese; CI, confidence interval; AUC, area under the curve.

**Table 8:** Multivariable regression analysis evaluating factors associated with weight loss.

Predictor	β (kg)	95% BCa CI	Bootstrap *p*
Mean CSI at 120 min	0.476	0.057 to 0.933	0.033
Baseline BMI	−0.172	−0.258 to −0.086	0.004
**Model performance**			
Statistic	Value		
R^2^	0.508		
Adjusted R^2^	0.447		
Bootstrap resamples	10,000		
Volunteers	19		

The dependent variable was body weight change from baseline to week 4. Regression coefficients are presented with bias-corrected and accelerated (BCa) bootstrap 95% confidence intervals based on 10,000 volunteer-level resamples.

## Data Availability

Please contact the corresponding author for data requests.

## Abbreviations

The following abbreviations are used in this manuscript:

HF	High-Fat
LF	Low-Fat
CSI	Combined Satiety Index
kg	Kilograms
GLP-1	Glucagon-like Peptide-1
PYY	Peptide YY
PUFA	Polyunsaturated Fatty Acids
CLA	Conjugated Linoleic Acid
HDL	High-Density Lipoprotein
BMI	Body Mass Index
GEE	Generalized Estimating Equations
VAS	Visual Analogue Scale
WHR	Waist–Hip Ratio
EMMs	Estimated Marginal Means
CI	Confidence Interval
AUC	Area Under the Curve
BCa	Bias-Corrected and Accelerated
